# Working toward a sustainable laboratory quality improvement programme through country ownership: Mozambique’s SLMTA story

**DOI:** 10.4102/ajlm.v3i2.253

**Published:** 2014-11-03

**Authors:** Jessina Masamha, Beth Skaggs, Isabel Pinto, Ana Paula Mandlaze, Carolina Simbine, Patrina Chongo, Leonardo de Sousa, Solon Kidane, Katy Yao, Elizabeth T. Luman, Eduardo Samogudo

**Affiliations:** 1 Center for Global Health (CGH), Division of Global HIV/AIDS (DGHA), US Centers for Disease Control and Prevention (CDC), Maputo, Mozambique; 2 CGH, Division of Global Disease Detection and Emergency Response, CDC, Atlanta, United States; 3 Central Laboratory Department, Ministry of Health, Maputo, Mozambique; 4 National Institute of Health, Ministry of Health, Maputo, Mozambique; 5 Association of Public Health Laboratories, Silver Spring, United States; 6 CGH, DGHA, CDC, Atlanta, United States

## Abstract

**Background:**

Launched in 2009, the Strengthening Laboratory Management Toward Accreditation (SLMTA) programme has emerged as an innovative approach for the improvement of laboratory quality. In order to ensure sustainability, Mozambique embedded the SLMTA programme within the existing Ministry of Health (MOH) laboratory structure.

**Objective:**

This article outlines the steps followed to establish a national framework for quality improvement and embedding the SLMTA programme within existing MOH laboratory systems.

**Methods:**

The MOH adopted SLMTA as the national laboratory quality improvement strategy, hired a dedicated coordinator and established a national laboratory quality technical working group comprising mostly personnel from key MOH departments. The working group developed an implementation framework for advocacy, training, mentorship, supervision and audits. Emphasis was placed on building local capacity for programme activities. After receiving training, a team of 25 implementers (18 from the MOH and seven from partner organisations) conducted baseline audits (using the Stepwise Laboratory Quality Improvement Process Towards Accreditation [SLIPTA] checklist), workshops and site visits in six reference and two central hospital laboratories. Exit audits were conducted in six of the eight laboratories and their results are presented.

**Results:**

The six laboratories demonstrated substantial improvement in audit scores; median scores increased from 35% at baseline to 57% at exit. It has been recommended that the National Tuberculosis Reference Laboratory apply for international accreditation.

**Conclusion:**

Successful implementation of SLMTA requires partnership between programme implementers, whilst effectiveness and long-term viability depend on country leadership, ownership and commitment. Integration of SLMTA into the existing MOH laboratory system will ensure durability beyond initial investments. The Mozambican model holds great promise that country leadership, ownership and institutionalisation can set the stage for programme success and sustainability.

## Introduction

Reliable laboratory services and networks are fundamental components of well-functioning health systems and are essential for patient management, disease detection and control, surveillance and outbreak investigations.^1^ However, in most resource-poor countries, Mozambique included, access to quality laboratory services is limited,^2^ resulting in delayed detection of outbreaks and lengthy or inaccurate diagnoses that may compromise patient care and outcomes. Laboratory quality improvement efforts are intended to strengthen laboratory services, leading to advances in the overall health system and the health of the nation.

**FIGURE 1:**
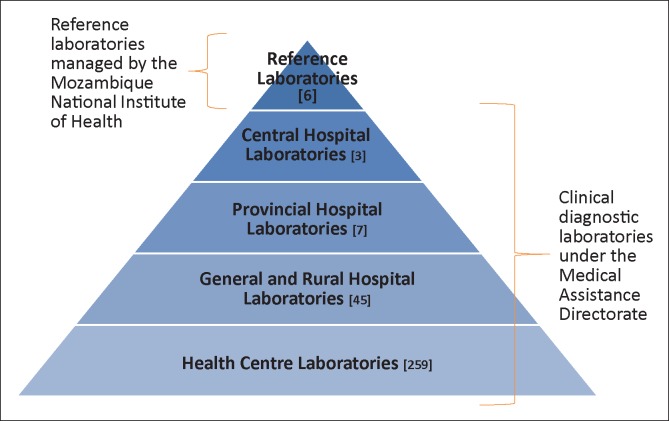
Tiered Ministry of Health laboratory network in Mozambique.

The national laboratory network in Mozambique consists of reference laboratories and clinical laboratories. Clinical laboratory services are integrated into a tiered National Health Service (NHS) that comprises central, provincial and district hospitals, as well as health centres (Figure 1). Of the more than 1300 health facilities in the NHS, only 314 (24%) have laboratories; approximately 30% of these offer only microscopy and rapid test services. Whilst strides have been made to improve laboratory infrastructure, a great number of health facilities and laboratories remain under-resourced in areas such as electricity, water supply, physical environment and equipment. In addition to constrained infrastructure, laboratories are affected by frequent stock outs of reagents and consumables, equipment breakdowns, insufficient numbers of qualified laboratory staff and a lack of laboratory quality systems.

Addressing gaps in the laboratory network is a critical objective for laboratory systems strengthening and a top priority for the Government of Mozambique.^3^ Consequently, the Ministry of Health (MOH) drafted a National Laboratory Policy that defines the governance structure for the national network in order to ensure consistent provision of quality laboratory services for clinical diagnosis, research and surveillance.^3^ The National Laboratory Policy outlines the Ministry’s commitment to the goals of implementing quality management systems (QMS) and pursuing national or international accreditation for its reference, central and provincial hospital laboratories.^3^

With these goals in mind, in 2010 the MOH in Mozambique implemented Strengthening Laboratory Management Toward Accreditation (SLMTA), an innovative training and mentorship programme for continuous quality improvement aimed at improving laboratory QMS and preparation for accreditation.^4^ In addition to SLMTA, the MOH adopted the World Health Organization Regional Office for Africa's (WHO AFRO) Stepwise Laboratory Quality Improvement Process Towards Accreditation (SLIPTA), a framework for measuring and recognising quality levels in public health laboratories in developing countries.^5^ SLIPTA incorporates a checklist to audit laboratory performance and a scoring system to determine a laboratory’s level on the pathway toward achieving accreditation to the International Organization for Standardization (ISO) 15189 standard.

The MOH incorporated SLMTA and SLIPTA into the existing health system infrastructure in order to overcome the challenges met by some externally-supported programmes that failed to last after support ended.^6^ Often, externally-supported programmes are viewed as short-term projects and therefore change is either resisted or temporary; desirable outcomes are either not achieved or not maintained. Institutionalisation, careful planning, local capacity building, country ownership and strong leadership have been described as being key ingredients for sustainable programmes.^7^ This article outlines the steps followed by the Mozambican MOH to establish a national framework for quality improvement and embed the SLMTA programme within existing MOH laboratory systems. Results from the initial cohort of participating laboratories are presented along with insights into strategies to establish programme sustainability.

## Research methods and design

### Institutionalisation of the SLMTA programme

In 2011, a national laboratory technical working group (TWG) consisting of key MOH personnel and partners was established to build a framework for a national laboratory quality improvement programme. The heads of Reference Laboratory Services and Clinical Laboratory Services within the MOH were appointed as co-chairs. The TWG developed a SLMTA implementation plan, which included training, mentorship, supervision and audits. Roles and responsibilities for each stakeholder were defined. The MOH created a logistics and administrative position under which the SLMTA coordinator was hired and a SLIPTA focal person was appointed. The MOH coordinated financing for programme implementation from its various partners. The TWG met weekly to coordinate and monitor the implementation of SLMTA. To ensure maximum programme integration and success, the TWG felt it was critical for SLMTA to be accepted as a national MOH programme within the laboratory network rather than as an externally-implemented project. To localise the programme, the SLMTA training tool kit was translated into Portuguese, locally-relevant implementation and advocacy strategies were developed and a Portuguese acronym, FOGELA (*Fortalecer a Gestão de Laboratórios para Acreditação*), was created. MOH TWG members were assigned leading roles in advocacy, planning and implementation. Prior to implementation, the SLMTA programme was introduced by TWG co-chairs through presentations at an annual National Health Directors meeting hosted by the MOH. Various members of the TWG introduced the programme at other meetings with key health leaders, including provincial health directors and medical directors in the provinces.

The SLMTA programme in Mozambique was designed to be implemented in a phased, hierarchical approach in which top-tier laboratories (national reference laboratories and central hospital laboratories) were prioritised for enrolment in the first cohort. After gaining experience implementing the programme in their own laboratories, trained managers and quality officers would themselves become a resource for training, mentoring and supervising laboratories enrolled in succeeding cohorts. After demonstrating competence in SLMTA implementation, provincial quality focal points would lead implementation at lower laboratory tiers within their provinces. To ensure feasibility, a small number of laboratories would be enrolled in successive years, prioritising provincial hospital laboratories. This phased approach was seen as essential for the development of SLMTA into a sustainable MOH programme that could keep pace with human and financial resource needs and maintain programme results.

Country ownership requires sufficient institutional capacity to define and implement a strategy.^7^ The need for trainers, mentors, supervisors and auditors was identified, and training was conducted in order to build the various skills required by MOH staff to successfully roll out and sustain the SLMTA programme. In December 2010, in collaboration with A Global Public Healthcare Foundation, Mozambique trained 15 auditors using the WHO AFRO Auditor Training curriculum. The following year, six of the auditors who were also TWG members were trained as SLMTA trainers, four from the MOH and two from the US Centers for Disease Control and Prevention (CDC) office in Mozambique. These SLMTA trainers also received training in mentorship and supervision and, as part of the TWG, oversaw programme implementation in the first cohort of laboratories. There was little in-country experience in implementing QMS at the inception of SLMTA. Thus, three expatriate laboratory professionals with experience in QMS development were contracted as mentors and trained by an experienced SLMTA mentor in the provision of guidance and support. In 2012, in preparation for programme decentralisation and expansion, Mozambique conducted the first Portuguese-language SLMTA Training-of-Trainers (TOT) course, which included 18 laboratory professionals from Mozambique and three from Angola. Mozambique now has a team of 25 SLMTA trainers, including four master trainers (two from the MOH) capable of conducting TOTs.

### Implementation

Eight laboratories were enrolled in the first SLMTA cohort, including six reference laboratories and two central hospital laboratories. In January 2011, newly-trained auditors were paired with the experienced consultant auditors to conduct baseline audits for the enrolled laboratories using the SLIPTA checklist. The checklist is divided into 12 sections. Laboratory quality is benchmarked on a zero- to five-star scale, awarded based on a percentage of the total score: < 55% assigned zero stars, 55% – 64% one star, 65% – 74% two stars, 75% – 84% three stars, 85% – 94% four stars and ≥ 95% five stars.^5^

The SLMTA curriculum was then presented during three 3–5-day workshops conducted four months apart. Three key staff members from each laboratory who were mandated to lead the implementation of quality improvement in their laboratory participated in the workshops: the laboratory manager, the quality officer and one head of section.

Throughout the programme, mentors worked with the laboratory staff, spending six- to eight-week periods embedded in a laboratory, followed by an eight-week absence. Selected improvement projects such as documentation, equipment management, inventory management, quality control and safety were implemented following each workshop. Two follow-up supervisory visits were conducted in each laboratory during these periods in order to provide technical assistance and to monitor the implementation process. The first visit was to support data collection for the selected improvement projects and to review laboratory action plans; the second visit six to eight weeks later was to monitor progress. Supervision reports were shared with participating laboratories and feedback on progress and challenges was shared with laboratory and hospital management.

In March 2012, after completion of the SLMTA curriculum, locally-trained auditors, supported by expert international auditors, conducted exit audits of the laboratories. Summaries from audit findings, corresponding scores and laboratory star ratings were shared with laboratory management and staff, hospital directors and provincial health department officials.

## Results

**FIGURE 2:**
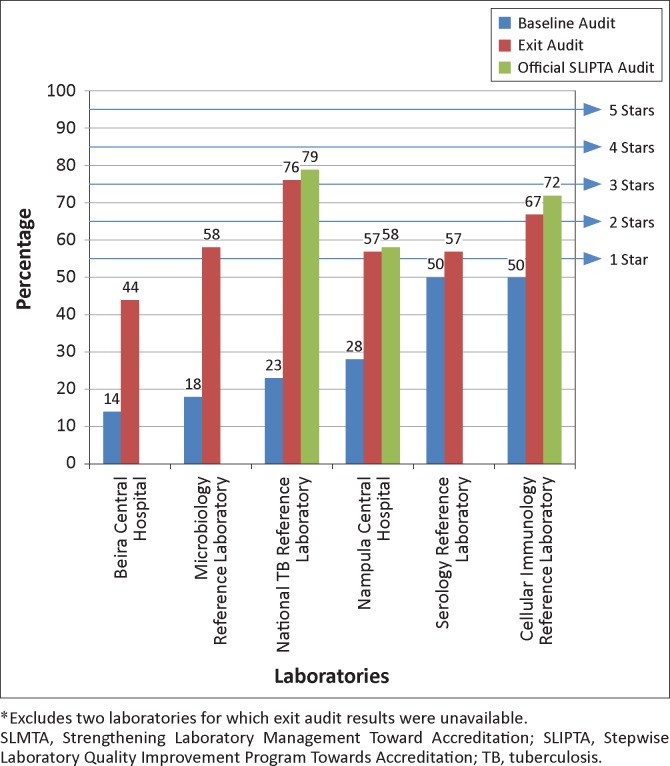
Baseline, exit and official SLIPTA audit results and star ratings for the first cohort^*^ of laboratories based on the SLIPTA checklist. Mozambique SLMTA programme, January 2011 to June 2013.

**FIGURE 3:**
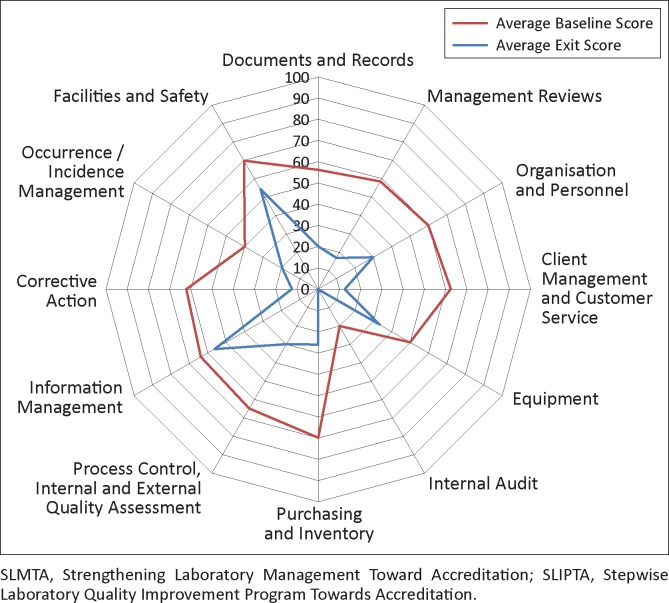
Average performance of six laboratories enrolled in first SLMTA cohort in Mozambique across the 12 sections of the SLIPTA checklist before and after SLMTA implementation as measured by baseline and exit audits (January 2011 to March 2012).

All eight laboratories completed the three SLMTA workshops and the assigned improvement projects within the implementation period. Of the eight laboratories, six had complete exit audit data; the remaining two reference laboratories had some missing data and were excluded from the analyses. The six laboratories with complete scores demonstrated overall improvement after 12 months of implementation (Figure 2). At baseline, all laboratories began with zero stars (median score 35%, range 14% – 50%). At exit, scores for all laboratories improved (median exit score 57%, range 44% – 76%; improvements 7–53 percentage points). One laboratory remained at zero stars (though its score increased from 14% to 44%), three laboratories were at one star, one laboratory was at two stars and one laboratory had reached three stars. The areas of greatest average improvement (> 40 percentage points) were client management and customer service, corrective action, purchasing and inventory and management reviews (Figure 3). The areas of least improvement (< 15 percentage points) were information management, equipment, facilities and safety and internal audit.

The best-performing laboratory at the exit audit was the National Tuberculosis (TB) Reference Laboratory, which attained three stars (76%). This laboratory had an overall improvement of 53 percentage points across all 12 checklist sections, with improvements of ≥ 80 percentage points in corrective action and purchasing and inventory.

At the end of the programme, three of the six laboratories were officially enrolled into the WHO AFRO SLIPTA programme for review by auditors from the African Society for Laboratory Medicine. The scores from the official audits were slightly higher than exit audit results, at 58% for Nampula Central Hospital, 72% for the Cellular Immunology Reference Laboratory and 79% for the National TB Reference Laboratory (Figure 2). The National TB Reference Laboratory was recommended to apply for international accreditation. A work plan was developed to address gaps identified and a target set for the laboratory to have the accreditation audit in August 2014 with the Portuguese Accreditation Institute based in Portugal.

## Discussion

Through SLMTA, Mozambique has begun to make substantial progress in laboratory quality improvement and has developed a plan to ensure that continued progress is both feasible and sustainable. The progress made would be short-lived without country ownership and leadership, both of which are fostered by institutionalisation of the programme. The MOH made a sustained effort to take ownership of the SLMTA programme as part of their overarching strategy to improve laboratory quality systems, embedding it within the MOH structure in order to ensure that planning, advocacy and implementation were led and championed by MOH personnel. As a result, laboratory management and staff viewed SLMTA as a permanent MOH programme, rather than as a ‘one-off’ event that would soon disappear.

MOH leadership not only influenced perception of the programme but also staff commitment. Laboratory personnel were wholly committed to the programme, working creatively and beyond designated work hours in order to implement assigned improvement projects. In one laboratory, the quality manager and laboratory staff contributed money to purchase file folders for laboratory documents. In another, laboratory personnel paid for tokens to reward staff who made outstanding contributions to quality improvement projects. In fact, so much excitement was generated over the programme that managers of laboratories yet to be enrolled in SLMTA wanted to know when their laboratories would be considered for future cohorts. The MOH led advocacy efforts, such as introduction of the programme and presentation of results at key strategic forums, which were aimed at promoting senior-level ownership, support and commitment crucial for the success of the programme. These efforts led to widespread buy-in and interest in the programme amongst provincial health and hospital directors as well as key MOH decision makers. The provincial and hospital directors received regular updates during routine SLMTA supervisory visits and showed a keen interest in knowing how their laboratories were progressing and what they could do to support the laboratory staff. Hospital directors began to visit the laboratories to follow up on projects and to ask ‘how many stars’ their laboratories had now achieved. These hospital directors commented on the increased interaction with the laboratories and improved understanding of their institutions’ quality indicators; and became involved in resolving problems that were beyond the laboratories' control. For example, when one laboratory showed decreased customer satisfaction as a result of staff shortages, the hospital director engaged the provincial health director to request additional laboratory staff at her facility.

As guided by the implementation plan, expansion of the SLMTA programme is underway. In October 2012, a second SLMTA cohort was launched. In keeping with the tiered strategy, laboratories from central, provincial, general and rural hospitals were eligible to apply and 10 laboratories were selected. As SLMTA roll-out continues, the laboratory TWG has set programme implementation goals based on the country’s capacity and available financial, human and material resources. The MOH continues to lead all aspects of programme planning and implementation and, since 2012, approximately 30% of funding for the programme has been incorporated into annual MOH budgets.

Mozambique has built a foundation for a sustainable laboratory quality improvement programme; however, to guarantee longevity, critical aspects of the national laboratory system must be strengthened. Establishing a National Laboratory Policy is essential with regard to sustaining the improvements achieved through the SLMTA programme.^1^ Whilst Mozambique has drafted such a policy, it has not yet been given official approval, limiting the MOH’s ability to drive the implementation of QMS and enforce adherence to quality standards across the laboratory network. Without such a formal policy, continued and sustained progress cannot be mandated systematically.

Mozambique has set a goal of accreditation for its six reference laboratories and 10 central and provincial hospital laboratories. However, international accreditation is not a goal that is achievable for all laboratories in the network. Thailand has successfully implemented a model to accredit laboratories according to national standards that vary by laboratory level.^8^ To ensure continuous quality improvement through expansion of SLMTA to lower-tier laboratories, Mozambique may consider developing a similar framework that outlines national standards for quality laboratory services, against which rural hospital and health centre laboratories could be monitored and evaluated. Once a policy is in place, clear measures must be established to assess progress and effectiveness, as well as to sustain laboratory strengthening efforts. Nkengasong et al. suggest using the indicator ‘number of laboratories accredited in the public health sector’ in order to track the progress of quality systems implementation for a national laboratory network.^9^ Additional indicators could include: number of provinces with dedicated quality management officers; percentage of laboratories audited in the previous 12 months; percentage of audited laboratories demonstrating improvement as measured by the SLIPTA checklist; and percentage of laboratories implementing external proficiency testing for select services.

Beyond system structures and policy, human resources are a critical requirement for the SLMTA programme. Yao et al. recommend an investment in human resources and allowance of time for dedicated personnel to successfully implement and sustain SLMTA activities so as to achieve the goal of laboratory accreditation.^4^ As Mozambique continues to work toward increasing coverage of SLMTA, it is critical to develop local training and mentoring capacity. Significant upfront investment in local capacity building will keep long-term costs down and ensure sufficient reach and uptake of the national programme. For example, in Zimbabwe, Shumba et al. found that it cost less over the long term to engage and train local quality managers as auditors, trainers and mentors than to import international consultants to do the job.^10^ Minimizing costs is essential because programmes that require unacceptable levels of resource commitment are unsustainable.^6^

### Challenges

Mozambique has invested in developing a large implementation team that has been trained adequately for the task. In fact, in addition to conducting in-country training, Mozambican-based trainers have since assisted with SLMTA training in Angola and Peru; whilst its master trainers have supported TOTs in Kenya, Ethiopia and the Dominican Republic. However, with the exception of the SLMTA logistics coordinator, none of these programme implementers work in an official capacity for the SLMTA programme. Because of competing priorities, SLMTA activities may not always be completed adequately, as evidenced by missing exit audit data for two laboratories in the first cohort. Within the laboratories, quality managers have bench responsibilities that compete with improvement projects and continued maintenance of the QMS. To be successful, the terms of reference for quality managers must be revised to include adequate time for implementation of the laboratory’s QMS. Although the initial plan was to groom local mentors from the first cohort of laboratories for subsequent cohorts, it has been a challenge to secure time for these now ‘more experienced’ staff members to leave their day-to-day work and provide mentorship to newly-enrolled laboratories. Staffing MOH quality departments with personnel dedicated to maintaining the daily operations of the quality improvement programme may address some of these challenges.

### Conclusion

Successful implementation of the SLMTA programme requires partnership; however effectiveness and long-term viability depend on country leadership, ownership and commitment. The MOH has collaborated with CDC and various partners to implement the SLMTA programme, whilst at the same time assuming leadership for the process, including establishment of the structures necessary for long-term programme success. The Mozambican model of institutionalisation of SLMTA into existing MOH laboratory systems demonstrates that country leadership, commitment and ownership can provide a strong platform for sustainability. By taking advantage of the current influx of laboratory resources to strengthen existing structures, build essential human resource capabilities and establish guiding strategies and policies, the Mozambique laboratory quality improvement programme is designed to be sustainable and is positioned to achieve the goal of providing quality laboratory services across the country.
